# A single capsule formulation of RHB-104 demonstrates higher anti-microbial growth potency for effective treatment of Crohn’s disease associated with *Mycobacterium avium* subspecies *paratuberculosis*

**DOI:** 10.1186/s13099-016-0127-z

**Published:** 2016-09-29

**Authors:** Ahmad Qasem, Mitra Safavikhasraghi, Saleh A. Naser

**Affiliations:** Burnett School of Biomedical Sciences, College of Medicine, University of Central Florida, 4110 Libra Drive, Orlando, FL USA

**Keywords:** *Mycobacterium paratuberculosis* (MAP), Crohn’s disease, RHB-104, MIC, Antibiotics, IBD

## Abstract

**Background:**

Most recently we reported that RHB‑104 triple antibiotics combination in culture is bactericidal and should be effective for treatment of Crohn’s disease (CD)-associated with *Mycobacterium avium* subspecies *paratuberculosis* (MAP) (Alcedo et al. in Gut Pathog 14:32, 2016). The combination exhibited unique synergistic antimicrobial growth activity. The proprietary RHB-104 capsule formulation contains active ingredients (63.3 % Clarithromycin (CLA), 6.7 % Clofazimine (CLO) and 30 % Rifabutin (RIF)). In our earlier study, we could not dissolve the proprietary RHB-104 capsule formulation in one compatible solvent. Consequently, we re-created RHB-104 analog by adding appropriate concentrations of each of the three antibiotics into the cultures. The Minimum inhibitory concentration (MIC) for RHB-104 analog, CLA, CLO, RIF, CLA-CLO, CLA-RIF, CLO-RIF and their individual solvents were reported earlier (Alcedo et al. in Gut Pathog 14:32, 2016). In this study, we succeeded in dissolving the proprietary RHB-104 capsule formulation in a single proprietary solvent. This study is designed to compare of the MIC the proprietary RHB-104 capsule formulation to RHB-104 analog against MAP and other microorganisms.

**Methods:**

BD Bactec™ MGIT™ Para-TB medium (Sparks, MD) system was used to determine the MIC of the proprietary RHB-104 capsule formulation and RHB-104 analog and their solvents against MAP and several other microorganisms. The final concentration of solvents used to dissolve all the drugs were ≤0.5 % (v/v).

**Results:**

The MIC for the RHB-104 proprietary solvent against MAP was consistent against all microorganisms tested in the study at 12.5 % (v/v). The MIC for the proprietary RHB-104 capsule formulation was similar to RHB-104 analog against several MAP clinical strains with MIC ≤ 0.2 μg/mL. The MIC for the proprietary RHB-104 capsule formulation was at 2.0 μg/mL against MAP strain MS 137 and *M. avium* strain JF7 compared to 4.0 ug/mL for RHB-104 analog. Similarly, the MIC of RHB-104 formulation capsule was significantly lower than RHB-104 analog against *M. tuberculosis* HR237, *M. fortuitism* subspecies *fortuitum, M. smegmatis* ATCC 27199, *Staphylococcus aureus* ATCC 25923 and *Listeria monocytogenes* ATCC 19112.

**Conclusion:**

The data demonstrated that the proprietary RHB-104 capsule formulation is more potent in culture against Mycobacteria and other microorganisms especially those with MIC >0.2. Formulation of multi-drugs in a single capsule results in potent synergistic anti-microbial activity far exceeds treatment the culture with multi-individually dissolved drugs. RHB-104 capsule formulation should be more effective to eradicate MAP infection in patients with CD. The study provides evidence that combining weak antibiotics in one formulation might be the new silver bullet to combat bacteria.

## Background

The current treatment guidelines of Crohn’s disease (CD) include immunosuppressants, anti-inflammatory drugs, nutritional therapy and antibiotics. *Mycobacterium avium* subspecies *paratuberculosis* (MAP) was isolated from intestinal tissues, milk and blood samples from CD patients at a higher frequency than controls [1–3]. Other microorganisms have been associated with CD such as *Listeria monocytogenes, Staphylococcus aureus, Klebsiella pneumoniae* and enteropathogenic *Escherichia coli* [4, 5]. CD patients treated with prolonged combination of macrolide-based anti-mycobacterial regimens in randomized clinical trials have achieved reversal of CD symptoms [6].

Most recently we evaluated the anti-microbial effect of three antibiotics regimen in the proprietary RHB-104 capsule formulation (RedHill Biopharma) in culture against 35 clinical MAP strains and other mycobacterial strains [7]. We determined the MIC for individual, and dual and triple combinations of the three antibiotics and their solvents [7] and have concluded that the triple combination of RHB-104 analog [a mixture of individually dissolved 63.3 % Clarithromycin (CLA), 6.7 % Clofazimine (CLO) and 30 % Rifabutin (RIF)] were more effective against MAP growth than when the cultures were treated with single or dual antibiotics dosages.

This study is focused on dissolving the proprietary RHB-104 capsule formulation in a solvent followed by determination of its MIC compared to RHB-104 analog against MAP and other microorganisms.

## Results

**Fig. 1:**
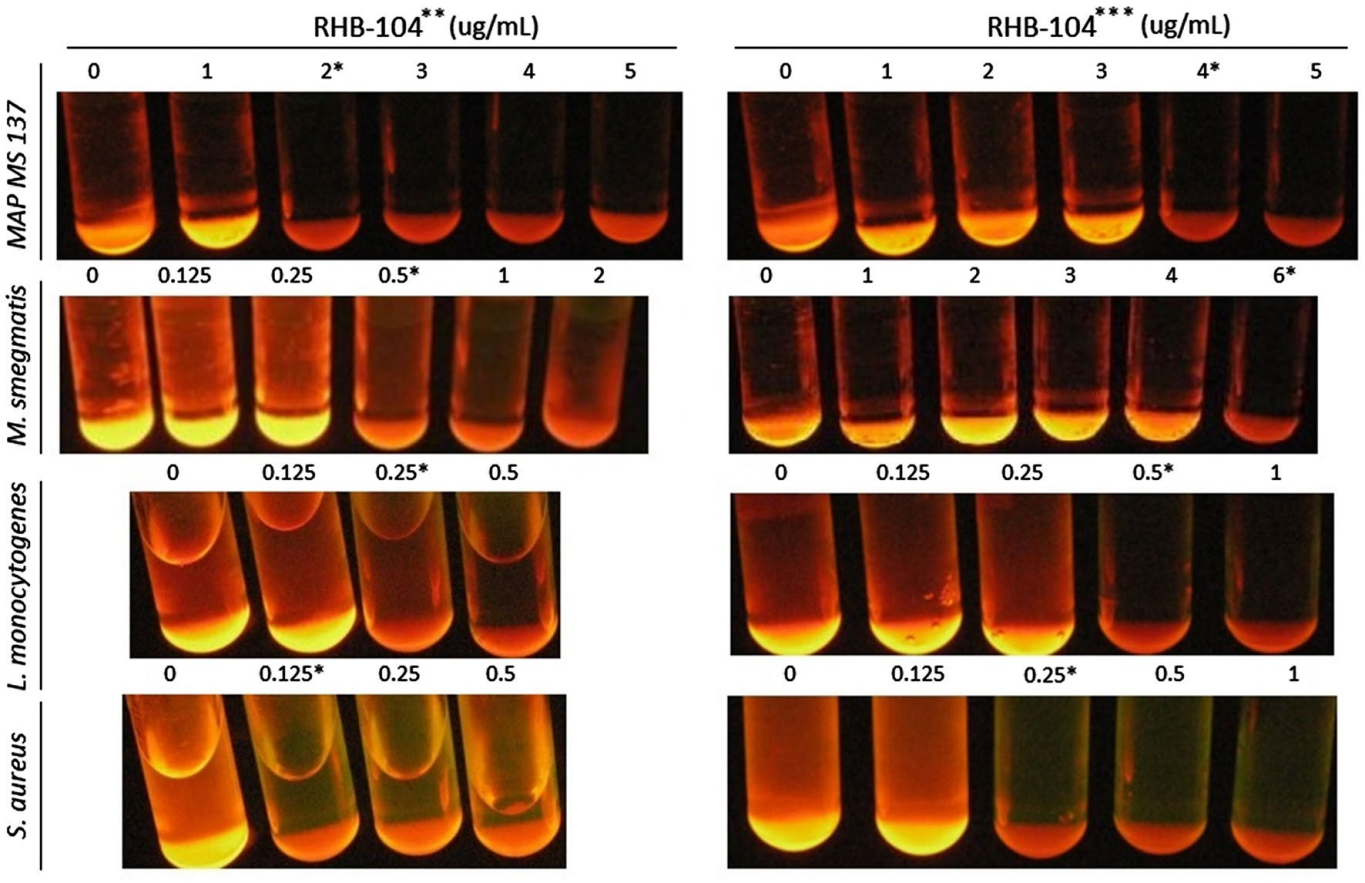
Effect of the proprietary RHB-104 capsule formulation and RHB-104 analog on culture growth of MAP *MS 137, M. smegmatis, L. monocytogenes* and *S. aureus*. * MIC level in μg/mL. ** The proprietary RHB-104 capsule formulation dissolved in one compatible solvent. *** RHB-104 analog (a mixture of individually dissolved 63.3 % Clarithromycin (CLA), 6.7 % Clofazimine (CLO) and 30 % Rifabutin (RIF), equal to  %composition in the proprietary RHB-104 capsule formulation)

In this study, we have successfully dissolved the proprietary RHB-104 capsule formulation in one compatible solvent. Due to proprietary ownership, RHB-104 capsule formulation solvent information can be requested directly from RedHill Biopharma. The MIC of the proprietary RHB-104 solvent against MAP and other microorganisms was at 12.5 % (v/v). The proprietary RHB-104 capsule formulation inhibitory effect on microorganisms with MIC ≤0.20 μg/mL was similar to RHB-104 analog (Fig. 1). However, the MIC for proprietary RHB-104 capsule formulation was at 2.0 μg/mL compared to 4.0 μg/mL for RHB-104 analog against MAP strain MS 137 from CD patient and *M. avium* strain *JF7* isolated from a HIV patient. The proprietary RHB-104 capsule formulation was more potent than RHB-104 analog against *M. fortuitum* subspecies *fortuitum* (MIC of 10 vs 15 μg/mL), *M. tuberculosis* strain HR237 (MIC of 5 vs 10 μg/mL), *M. smegmatis* ATCC 27199 (MIC of 0.5 vs 6 μg/mL), *S. aureus* ATCC 25923 (MIC of 0.125 vs 0.25 μg/mL), and *L. monocytogenes* ATCC 19112 (MIC of 0.25 vs 0.5 μg/mL). Interestingly, the MIC for CLA (63 % of the proprietary RHB-104 capsule formulation) against *S. aureus* and *L. monocytogenes* was at 1.5 μg/mL. Table 1 summarizes MIC values for both the proprietary RHB-104 capsule formulation and RHB-104 analog against several microorganisms used in this study. There was no significant inhibitory effect in culture on the growth of *K. pneumonia* (ATCC 13883) and plasmid-harboring *E. coli (*MIC >40 μg/mL). The final concentration of solvents used to dissolve all the drugs in the study were ≤0.5 % (v/v) and had no effect on bacterial growth in cultures.

**Table 1:** MIC of the proprietary RHB-104 capsule formulation and RHB-104 analog

Microorganism	Minimum inhibitory concentration (μg/mL)	
	CLA-CLO-RIF (RHB-104 analog)^a^	Proprietary RHB-104 capsule formulation^b^
*MAP MS 137*	4.0	2.0
*M. avium—JF7*	4.0	2.0
*M. fortuitum ss fortuitum*	15	10
*M. tuberculosis* HR237	10	5.0
*M. smegmatis* ATCC 27199	6.0	0.125
*S. aureus* ATCC 25923	0.25	0.125
*L. monocytogenes* ATCC 19112	0.5	0.25
*K. pneumonia* ATCC 13883	>40	>40
Recombinant *E. coli*	>40	>40

All cultures were performed in duplicates and have been repeated three times

^a^RHB-104 analog where the 3 drugs dissolved individually were combined in one solution at their proprietary RHB-104 capsule formulation composition percentage

^b^Proprietary RHB-104 capsule formulation was dissolved in one compatible solvent

## Conclusion

The ability to evaluate the proprietary RHB-104 capsule formulation dissolved in one compatible solvent against broad spectrum of microorganisms demonstrated that this investigational single capsule formulation is more potent against *Mycobacteria* and other microorganisms associated with CD than previously determined. The study clearly concludes that the synergistic effect between CLA, CLO and RIF triple formulation in a single capsule is strong and therefore, favoring formulation-approach treatment. The data also suggests that the formulation approach might be used to re-evaluate antibiotics that have been declared as ineffective when used individually. The study strongly suggest that the proprietary RHB-104 capsule formulation should be more effective to eradicate MAP infection than regimens with multi-individualized antibiotics. Ultimately, one single capsule formulation treatment should enhance compliance and clinical outcome for CD patients.

## Abbreviations

CD: Crohn’s disease
MAP: *Mycobacterium avium* subspecies* paratuberculosis*
CLA: clarithromycin
CLO: clofazimine
RIF: cifabutin
MIC: minimum inhibitory concentration
RHB-104: RedHill Biopharma drug #104
